# The Intersection of COVID-19 and Autoimmunity: What is Our Current Understanding?

**DOI:** 10.20411/pai.v6i1.417

**Published:** 2021-03-08

**Authors:** N. Winchester, C. Calabrese, L.H. Calabrese

**Affiliations:** 1 Cleveland Clinic Lerner College of Medicine of Case Western Reserve University, Cleveland, OH; 2 Department of Rheumatic and Immunologic Diseases, Cleveland Clinic, Cleveland, OH

**Keywords:** COVID-19, SARS-CoV-2, Autoimmunity, Immune-mediated inflammatory disease, MIS-C, Autoantibodies, DMARDs

## Abstract

Viral infections have historically had a complex relationship with autoimmune diseases. For patients with preexisting autoimmune disorders, often complicated by immunosuppressive therapies, there are numerous potential effects of COVID-19, a disease of complex immunobiology, including the potential for an altered natural history of COVID-19 when infected. In addition, individuals without recognized autoimmune disease may be vulnerable to virally induced autoimmunity in the forms of autoantibody formation, as well as the development of clinical immune-mediated inflammatory diseases. Until quite recently in the pandemic, this relationship between COVID-19 and autoimmune diseases has been relatively underexplored; yet such investigation offers potential insights into immunopathogenesis as well as for the development of new immune-based therapeutics. Our review examines this relationship through exploration of a series of questions with relevance to both immunopathogenic mechanisms as well as some clinical implications.

## INTRODUCTION

### a) Viruses and autoimmunity

Viral infections and autoimmune diseases have a complex relationship. Our antiviral defenses against most viral pathogens represent an integrated response from both innate and adaptive immunity. This includes a prominent role of interferon (IFN), the elaboration of specific humoral and cellular responses, the production of effectors of inflammation and repair, including an array of cytokines and chemokines, and immunoregulatory elements capable of bringing the immunologic attack to an end with the development of immunologic memory [1]. These same effector pathways also contribute to the immunopathogenesis of autoimmune and autoinflammatory diseases, which arise when there is an imbalance between effector inflammatory pathways and tolerogenic control mechanisms favoring the inflammatory response [2]. The nature of this imbalance remains incompletely understood for most immune-mediated diseases. In general, autoimmunity is believed to result from a combination of genetic and environmental factors, among which may include viral infections [2, 3]. Numerous viral illnesses have been well documented to be etiologic for a variety of autoimmune diseases, including hepatitis C and cryoglobulinemia [4], hepatitis B and arthritis and vasculitis [5], HIV and a series of inflammatory disorders [6], enteric viruses and Type 1 diabetes [7], and herpes viruses with a variety of autoimmune diseases including systemic lupus erythematosus (SLE), rheumatoid arthritis (RA), Sjögren's disease, and others [3, 8]. The mechanisms underlying these associations remain poorly understood, but candidates with varying levels of evidence suggest a role for molecular mimicry, bystander activation, breaching of central or peripheral tolerogenic pathways, aberrant nucleic acid sensing, and others [1, 2, 9]. In general, immunologic diseases are classified as either autoimmune or autoinflammatory. In autoimmune disease (eg, SLE, RA) there is evidence of an adaptive response against self (ie, specific antibodies or cellular responses to host antigens). In autoinflammatory disease there is no or limited evidence of such reactivity, and the inflammation is mediated by dysregulated innate immune responses (eg, Behçet's, cryopyrinopathies). These diseases have been proposed to represent a spectrum of immune dysregulation with many clinical entities demonstrating varying degrees of overlap between autoimmunity and autoinflammation [10]. Collectively, these disorders are often referred to as immune-mediated inflammatory diseases or IMIDs, a term which we will use for this review.

In December of 2019 the first cases of a disease (COVID-19) caused by the coronavirus SARS CoV-2 were reported in Wuhan, China. Since that time, we have experienced a global pandemic and a rapid surge in biotechnological research in an effort to understand, manage, and prevent the disease. Because infections with SARS CoV-2 have the capacity for a robust inflammatory phase involving pathways and mediators well known as effective clinical targets in many autoimmune or autoinflammatory diseases, it is reasonable to examine the interrelationship between COVID-19 and IMIDs. This narrative review is an attempt to critically appraise the intersection of COVID-19 and IMIDs by examining a series of questions that are of current interest in the field. We will focus on select areas such as epidemiologic data from international registries of IMID patients with COVID-19, newly described syndromes with features of IMIDs such as the multisystem inflammatory disease of children (MIS-C) [11], the multisystem inflammatory disease of adults (MIS-A) [12], post COVID-19 syndromes (long COVID-19) [13], and some recent work of note in basic immunobiology that has explored the underlying mechanisms of autoimmunity in COVID-19.

**Figure 1.**
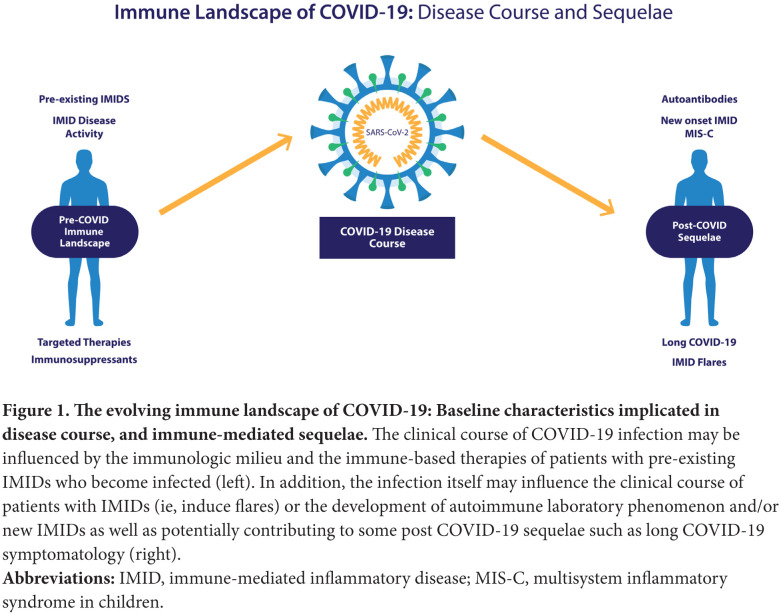
The evolving immune landscape of COVID-19: Baseline characteristics implicated in disease course, and immune-mediated sequelae. The clinical course of COVID-19 infection may be influenced by the immunologic milieu and the immune-based therapies of patients with pre-existing IMIDs who become infected (left). In addition, the infection itself may influence the clinical course of patients with IMIDs (ie, induce flares) or the development of autoimmune laboratory phenomenon and/or new IMIDs as well as potentially contributing to some post COVID-19 sequelae such as long COVID-19 symptomatology (right). **Abbreviations:** IMID, immune-mediated inflammatory disease; MIS-C, multisystem inflammatory syndrome in children.

The relationships between IMIDs and COVID-19 can be viewed bidirectionally (Figure 1). On the left are those patients with IMIDs who develop COVID-19. For these individuals who have perturbed immune systems to begin with, there are numerous unanswered questions including how their underlying disorders of immunity, as well as the immunomodulatory therapies they are receiving, may affect the clinical course of COIVD-19 once infected. Examining this experience could potentially provide insights into the role of specific immune pathways contributing to the pathogenesis of COVID-19, similar to understanding the outcomes of infections in patients with inborn errors of immunity [14].

To the right are post-COVID-19 sequelae, where we can explore the evidence that COVID-19 itself can induce autoimmunity or autoinflammation, potentially leading to autoimmune laboratory phenomena or frank IMIDs or IMID-like conditions, intercurrent with, or following infection. This has recently become more important as a variety of late complications of COVID-19 have been described including several delayed inflammatory syndromes. These include MIS-C [11], MIS-A [12], and a poorly defined spectrum of clinical sequelae of unclear pathogenesis now often referred to as long COVID-19 [13] Wingdings. These delayed disorders are still poorly understood but appear to be post infectious phenomena, and a role for virus-induced immune dysregulation has been proposed as being potentially etiogenic [15, 16]. We will address this complex topic via a narrative review wherein we attempt to answer a series of questions based on the current data.

### b) General concepts – immunopathogenesis of COVID-19

**Figure 2.**
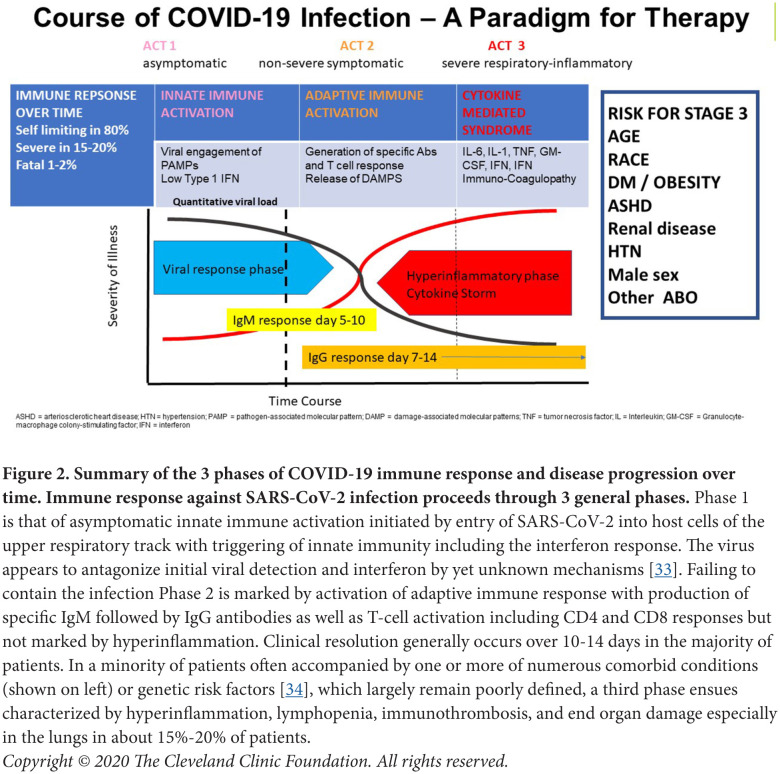
Summary of the 3 phases of COVID-19 immune response and disease progression over time. Immune response against SARS-CoV-2 infection proceeds through 3 general phases. Phase 1 is that of asymptomatic innate immune activation initiated by entry of SARS-CoV-2 into host cells of the upper respiratory track with triggering of innate immunity including the interferon response. The virus appears to antagonize initial viral detection and interferon by yet unknown mechanisms [33]. Failing to contain the infection Phase 2 is marked by activation of adaptive immune response with production of specific IgM followed by IgG antibodies as well as T-cell activation including CD4 and CD8 responses but not marked by hyperinflammation. Clinical resolution generally occurs over 10-14 days in the majority of patients. In a minority of patients often accompanied by one or more of numerous comorbid conditions (shown on left) or genetic risk factors [34], which largely remain poorly defined, a third phase ensues characterized by hyperinflammation, lymphopenia, immunothrombosis, and end organ damage especially in the lungs in about 15%-20% of patients.

To better understand the interrelationships between COVID-19 and IMIDs, it is useful to summarize the idealized immunopathogenesis of COVID-19, recognizing that it is a rapidly changing field (Figure 2) [17]. What has become clear is that in the majority of individuals, infection with SARS-CoV-2 is mild, including asymptomatic and pauci-symptomatic forms, and these persons successfully defend themselves from the virus via triggering of innate immunity (Phase I). This is characterized by an early IFN response and then, over time, by induction of an adaptive immune response (Phase 2) with specific immunoglobulin production accompanied by T-cell activation and the generation of memory cells [18, 19]. For a minority of infected individuals, however, the disease is severe, especially for those with numerous comorbidities, who then may progress to pneumonitis with yet a smaller percentage experiencing critical complications (acute respiratory failure, shock, immunothrombosis, multiorgan dysfunction or failure, and death) (Phase 3) [20]. This third phase is accompanied by an immune response characterized by hyperinflammation, reminiscent of a wide number of disorders often collectively referred to as cytokine release or cytokine storm, although accepted definitions for these syndromes are lacking across the wide spectrum of disorders in which they are observed [21, 22]. The underlying immunopathologic mechanisms contributing to this state of hyperinflammation remain unclear, but several recurrent themes have been noted. First, numerous groups have documented defects in type 1 and 3 IFN responses early in infection [23, 24], suggesting defects in viral control. Second, the hyperinflammatory state itself is characterized by a rich signature of inflammatory biomarkers indicative of dysregulated innate and adaptive limbs of immunity [25, 26]. Among the many issues that remain unclear include our understanding of the defects in counter regulatory pathways that allow this state of hyperinflammation to persist, as well as understanding the underlying drivers of this final inflammatory phase of the infection. Some evidence suggests that it may be pathogen driven, as several groups have documented increased viral loads in those with the most severe forms of disease [27, 28, 29]. Others have emphasized a dyssynchrony between immune events occurring in the lung and those studied in the blood and have suggested that damage-associated products (ie, DAMPS) may be preferential drivers of hyperinflammation, particularly in the setting of advanced pneumonitis [30]. The role of host factors has also been noted, including subclinical immune deficiency states associated with silent heterozygous gene variants, reflecting defects in type I IFN signaling [31], as well as the propensity of some to develop natural autoantibodies to Type I IFN [32].

Copyright © 2020 The Cleveland Clinic Foundation. All rights reserved.

Improved understanding of immunopathogenesis has profound implications on potential therapies. The landscape of treatment for COVID-19 is rapidly evolving with over 2500 clinical trials currently registered (https://www.covid-trials.org/, December 30, 2020). Many of the therapeutics under investigation are repurposed from the formidable armamentarium of immunomodulatory therapies used for patients with immune-mediated disorders, including targeted therapies directed at cytokines (TNF, IL-1, IL-6, GM-CSF, IL-23, and others), kinase inhibitors (Janus kinase, Bruton's kinase, and others), and broad immunomodulatory therapies such as glucocorticoids [35–38]. A critical examination of the clinical outcomes of patients with IMIDs and with COVID-19 who are already receiving these targeted therapies provides a unique opportunity to assess their potential role as therapeutics for the general non-IMID population.

## CRITICAL QUESTIONS

### What do we know about the clinical course of patients with IMIDs who develop COVID-19?

Several critical questions remain unanswered regarding patients with pre-existing IMIDs and COVID-19, including whether they may be more susceptible to infection, and whether they may experience more severe disease once infected. Both of these are meritorious but difficult to assess. Attempts to address these questions are confounded by the nature of the investigations and data sources and the heterogeneity of IMIDs themselves. Even if one focuses on major categories of IMIDs such as rheumatic disorders (inflammatory arthritis, connective tissue diseases, vasculitis, etc), inflammatory bowel disease, psoriasis, and multiple sclerosis, the task is challenging given that these disorders have divergent immunopathogenic mechanisms limiting the strength of any conclusions. Numerous small studies have attempted to assess the risks of IMIDs (rheumatic diseases, inflammatory bowel disease, psoriasis, multiple sclerosis) in patients who acquire COVID-19, and none to our knowledge have demonstrated any such propensity [39, 40, 41, 42]. Strong conclusions should not be inferred, for patients with IMIDs may not be as exposed, as in small investigations they have been shown to practice greater social distancing and greater general precautions, possibly based on such concerns [43, 44]. To appraise how patients with IMID progress once they develop COVID-19, we are fortunate to have a growing number of registries designed to collect data specifically on IMIDs and COVID-19 such as the COVID-19 Global Rheumatology Alliance (CGRA) (includes all inflammatory rheumatic diseases) [45], the Secure IBD registry (includes all forms of inflammatory bowel disease) [46], the PsoProtect registry (psoriasis) [47], and the MS Data Alliance (Multiple Sclerosis) [48], among others. All of these registries are limited by design, including that they are voluntary, are subject to reporting bias, have low granularity of data, and lack comparator non-disease control subjects, leaving them vulnerable to numerous confounders. Despite these limitations, the registries are powerful because of their size.

Another important question is whether patients with IMIDs, specifically rheumatic, IBD, psoriasis and MS patients with COVID-19, have more severe outcomes. These data are derived largely from numerous small case-controlled investigations. In general, patients with IMIDs such as rheumatic diseases [49], IBD [50], and multiple sclerosis [42] appear to be more vulnerable to severe outcomes (ie, hospitalization, ICU status, and death), but after adjusting for comorbid conditions that are overrepresented in patients with autoimmune disease, such as cardiovascular disease, this risk is greatly diminished in most [49], but not all investigations. Preliminary data, primarily derived from case reports and small case studies have also associated SARS-CoV-2 infection with disease flares in patients with pre-existing IMIDs, but conclusions of causation should be avoided [51]. Disruptions in rheumatology care during the pandemic and inconsistent guidelines for withholding immunomodulatory therapies following SARS-CoV-2 infection make these findings difficult to interpret.

### What lessons may be learned from examining the clinical outcomes of patients on immunomodulatory therapy at baseline?

An important area of investigation is the opportunity to examine patients with IMIDs on targeted or broad immunosuppressive therapy at the onset of COVID-19 to assess their potential effect on clinical course. One consistent finding from both the CGRA data set, numbering over 7000 patients, and the Secure-IBD data base, numbering over 4000 subjects as of December 30, 2020, is a significant risk of severe outcomes in patients receiving a daily moderate to high glucocorticoid dosage (prednisolone-equivalent dosage of >10mg/day) [52]. It is presumed that glucocorticoids may be generally immunosuppressive and thwart robust innate and adaptive immune responses based on their broad immunomodulatory mechanism of action [53]. Even more potentially informative is the examination of the clinical outcomes of COVID-19 in patients with IMIDs who acquire the infection while already on biologic disease-modifying antirheumatic drugs (DMARDs), in particular anti-cytokine agents, as well as targeted synthetic DMARDs (ie, JAK inhibitors and others). In these settings the effects of select immune inhibition early in the course of infection may provide unique opportunities for insights not possible when such agents are not started until the disease progresses. The CGRA is the largest COVID-19 specific registry among IMIDs and has revealed that having a clinically uncontrolled IMID, as well as not receiving immunosuppressive therapy, are risks for severe outcomes [54]. In terms of specific therapies, aside from glucocorticoids, receiving rituximab or sulfasalazine is associated with higher odds of death, compared to methotrexate monotherapy (OR 4.04, 95% CI 2.32-7.03) and (3.60, 1.66-7.78), respectively [54]. This significant association with sulfasalazine is surprising given that it is considered a weak immunomodulatory agent, but a similar finding has been demonstrated in the SECURE–IBD registry [55], and thus should serve as a hypothesis-generating observation. Alternatively, receiving anti-cytokine therapy (TNF inhibitors or IL-6 inhibitors) at onset of infection was not associated with severe outcomes, and in the CGRA registry, TNF inhibition was associated with decreased risk of hospitalization [56]. In the PsoProtect study, patients with psoriasis taking TNF inhibitors also demonstrated more favorable outcomes from COVID-19 [57], and similar trends were noted in the SECURE-IBD registry [50]. Such observations may be hypothesis generating and serve as a rationale for targeted therapy of COVID-19 at early time points in the infection. Data on other drugs of interest such as Janus kinase inhibitors, abatacept, IL-17 inhibitors, and others are limited due to small numbers, but will no doubt increase over time.

### Does COVID-19 induce autoantibodies and autoimmune disease?

As discussed in the introduction, several viral infections are known to induce autoantibodies and clinical IMIDs, and growing evidence implicates SARS CoV-2 in these processes (Figure 1). However, numerous questions remain unanswered, including whether autoantibodies produced during infection with SARS CoV-2 are epiphenomena or drivers of clinical disease, and what are the underlying mechanisms responsible for their production. Numerous studies have documented the presence of a wide variety of autoantibodies in patients with COVID-19, with antinuclear antibody (ANA) reported in up to 40%-50% of severe cases [58, 59], and anti-SSA/Ro [58], rheumatoid factor (RF) [59], and antibodies against IFN-I [32] reported in up to 25%, 19%, and 10% of patients, respectively. However, at this time it is unclear if these antibodies are linked to clinical autoimmunity, or even result from COVID-19, and if so, through what mechanisms. The presence of autoantibodies in COVID-19, specifically ANA and RF, has been correlated with CRP levels [59] and shown to be higher overall than in uninfected controls, with targets involving immunomodulatory proteins including cytokines, chemokines, complement factors, and cell surface proteins [60]. A recent study supported a link between autoantibodies and poor COVID-19 outcomes. The study found that the level of autoantibodies against the protein annexin A2, responsible for cell membrane stabilization, was significantly higher in patients who died of COVID-19 than those who survived [61]. While these findings suggest a detrimental role of autoantibodies in the course of COVID-19, drawing strong clinical correlations will require more data. Furthermore, given the designs of these investigations, it is difficult to determine if these autoantibodies are the result of infection with SARS-CoV-2, or are pre-existing. A further consideration yet unproven is that such autoantibodies may instead drive a symptomatic disease course in individuals who later become infected.

Immunocoagulopathy, so frequently encountered in severe COVID-19, has been correlated with high inflammatory biomarkers and the detection of antiphospholipid (aPL) antibodies in numerous case reports, suggesting that they may play a role in pathogenesis [62]. Development of aPL antibodies (lupus anticoagulant [LAC], anti-cardiolipin [aCL], and anti-β2-glycoprotein I [a β2GPI]), has been reported at varying frequencies following other viral infections, most notably HIV and HCV, but do not always co-occur with thromboembolic events, and can be found at modest levels in the general population [63]. Antiphospholipid antibodies as a class have been reported at the highest frequency of all autoantibodies, detected in about half of severe cases [64] and being highest in those in the ICU, affecting up to 91% of COVID-19 patients with a prolonged activated partial thromboplastin time (aPTT) [65], but their mechanistic role in clotting and clinical significance has been unclear until recently. Revealing work by Knight and colleagues detected aPL antibodies in about half of patients hospitalized with COVID-19, and found that the presence of neutrophil extracellular traps (NETs), which are prothrombotic in antiphospholipid syndrome (APS), were associated with higher titers of aPL antibodies in patients with COVID-19 [64]. IgG fractions purified from patients with severe COVID-19 were furthermore shown to accelerate thrombosis when injected into mice, as demonstrated in other studies of APS. These findings reveal a potential role of aPL antibodies in potentiating thrombosis in hospitalized patients with COVID-19 through promotion of NET formation and highlight aPL antibodies and NET formation as appealing therapeutic targets. Clinical trials to evaluate the potential therapeutic effect of inhibiting NETosis, or promoting degradation of NETs, are urgently needed.

In addition to autoreactive antibodies, there are also case reports of clinical autoimmune diseases post SARS-CoV-2 infection, including autoimmune cytopenia, immune thrombocytopenic purpura, Guillain-Barre syndrome (GBS), Miller Fisher syndrome, and acute disseminated encephalomyelitis [8, 66]. While these case reports are informative, they are limited by reporting bias, and it is difficult to determine if these patients would have otherwise developed IMIDs without SARS-CoV-2 infection, and therefore whether these findings are coincidence, rather than causation. A recent study sought to investigate this relationship between SARS-CoV-2 and GBS in the UK, and found that the incidence of GBS had declined during the pandemic, suggesting that SARS-CoV-2 is either not a trigger for GBS in this population, or it is causing fewer cases than are being prevented by the lockdown and new hygiene practices [67]. However, the ability of viral infections to trigger autoimmunity, reports of molecular mimicry between SARS-CoV-2 and human tissues [68], and onset of autoimmunity within a month of first COVID symptoms [66] suggest SARS-CoV-2 may be etiologic for other types of IMIDs, or for GBS in other genetic backgrounds. At present, insufficient data exist to delineate a role of COVID-19 in triggering new onset IMIDs.

### What is the relationship between autoimmunity and the newly defined post COVID-19 syndromes such as MIS-C and MIS-A?

In addition to classical autoimmune disease reported following COVID-19, several novel hyperinflammatory syndromes have been described during the pandemic, including chilblain-like lesions (a small vessel inflammatory vasculopathy also known as “COVID toes”), multi-system inflammatory syndrome in children (MIS-C), and a similar syndrome in adults (MIS-A). These syndromes have been temporally associated with SARS-CoV-2 infection, following viral clearance, raising questions about the role of a post-viral process, whether they result from an immune inflammatory or autoimmune process triggered by COVID-19, and whether they are truly novel syndromes or are presentations of previously described post-viral inflammatory syndromes. Reports of delayed onset chilblain-like lesions predominantly affecting the lower extremities have increased during the pandemic, but strong conclusions about its etiology cannot be made due to inconsistent clinical and laboratory evidence of prior SARS-CoV-2 infection. While the younger age of these patients make asymptomatic or mild COVID-19 more likely, and may therefore reduce the sensitivity of SARS-CoV-2 testing, no study has proven a pathologic link between SARS-CoV-2 and chilblains [69]. Results of a recent study suggest that chilblains may be a manifestation of a post-viral type I interferonopathy [69]. This is interesting as similar vascular lesions are observed in other disorders characterized by upregulation of type I IFNs such as STING-associated vasculopathy of infancy [70] [69], but further research is needed to definitively link SARS-CoV-2 with such mechanisms.

Since April 2020, over 1200 cases have been reported of a novel, life-threatening syndrome now termed MIS-C in children following COVID-19. MIS-C is characterized by fever, multisystem organ involvement, and significantly elevated inflammatory biomarkers [71]. Early reports noted similarities between the clinical presentation of MIS-C and Kawasaki Disease (KD), leading to speculation of an autoinflammatory process. Elevated inflammatory biomarkers in MIS-C and presentation of symptoms after SARS-CoV-2 clearance are both congruent with an autoinflammatory or autoimmune, rather than infectious, process [72]. This is further supported by the rapid clinical improvement in these children when treated with immunomodulatory and anti-inflammatory drugs [72]. However, data on the disease course without such intervention is sparse. Our understanding of the immunopathogenic mechanisms underlying MIS-C is rapidly evolving, and suggests an aberrant immune landscape in MIS-C, distinct from that of severe acute SARS-CoV-2 infection, and perhaps KD. The current evidence implicates aberrant innate immunity in MIS-C, with reports of increased complement activation [73], activated neutrophils and monocytes [74], and elevated pro-inflammatory cytokines and markers of inflammation [75]. The potential roles of adaptive immunity and autoantibodies have also garnered considerable interest; classical autoantibodies, and antibodies against self-peptides (endothelial, cardiac, and gastrointestinal), have been identified in MIS-C [75]. These findings collectively show robust activation of innate and adaptive arms of immunity in MIS-C that could either be drivers of an IMID process, or consequences of it.

Comparisons have been made between KD and MIS-C, as the clinical presentations overlap, and both have been associated with viral infections. While this is hypothesis generating for the immunopathogenesis of MIS-C, KD is still incompletely understood, and it is unknown if they represent variants of a single syndrome. Key epidemiologic differences, with MIS-C affecting older black and Hispanic children, and KD affecting young children of Asian ethnicity, have led to speculation that these represent unique disease processes, with differences in genetic predisposition for each, and a potential role of pre-existing immune memory in driving MIS-C [76]. A recent study showed higher markers of arterial damage in KD, congruent with its predilection for the arteries, and higher markers of cardiac stress in MIS-C, where shock and cardiac dysfunction are more frequent [72]. Deep immune profiling in another cohort identified higher levels of activated and proliferating cytotoxic T cells in MIS-C that home to the vasculature and normalize with clinical improvement [77]. These select findings highlight distinct features of KD and MIS-C, however many similarities exist, and it remains to be determined if MIS-C and its etio-pathogenesis is distinct from KD or is part of the clinical spectrum.

Recently, the CDC proposed criteria for a similar syndrome in adults, termed MIS-A, characterized by hyperinflammation in the absence of severe pulmonary disease, and suggested it resulted from an autoinflammatory response to SARS-CoV-2 infection [12]. While the pathophysiology of MIS-A remains poorly understood, comparisons of the immune landscape and clinical characteristics of KD, MIS-C, and MIS-A may help clarify the immunopathogenesis of each, and lead to more informed management.

### Do we understand the mechanisms of autoimmunity in COVID-19?

Our current understanding of the underlying mechanisms for systemic and organ-specific autoimmunity is incomplete. In terms of research output most work has focused on the phenotype, and more recently on the underlying immunologic endotype, of the hyper-inflammatory phase 3 of COVID-19 often referred to as a cytokine storm or cytokine release syndrome. This has been extensively reviewed [22, 25, 78, 79]. The precise role of generalized inflammation as a driver of systemic and organ-specific autoimmunity via bystander immune activation has been proposed [80]. More recently an exciting area of work has focused on the similarities between the B-cell immunophenotypes in Phase 3 COVID-19 and patients with active systemic lupus erythematous (SLE). Work by Sanz and colleagues [81] has previously characterized a unique group of IgD and CD27 double-negative (DN) B cells in SLE, associating the CXCR5-CD21-CD11c+ (DN2) subset with increased disease activity and poor clinical outcomes. These cells are indicative of an extra-follicular origin of B-cell maturation and are capable of making autoantibodies [81]. This same group, using high-dimensional B-cell analysis, has documented a similar cell population in patients with advanced COVID-19 correlating their presence with inflammatory markers such as CRP and IL-6, which are characteristic of phase 3 COVID-19, suggesting shared pathways between a prototypic autoimmune disease and the hyper inflammatory phase of COVID-19 [81, 82]. In separate work Pillai and colleagues [83] have documented that germinal centers are lost in the lymph nodes and spleens of patients with acute COVID-19 and that aberrant TNF alpha production may underlie this anatomic destruction. This work supports a model suggested by the observations of Sanz and colleagues wherein an anatomic driver of extrafollicular B-cell maturation drives disease and furthermore suggests that anti-TNF may be a logical therapeutic to potentially prevent this. These select studies on the intersection of autoantibodies and COVID-19 immunopathogenesis have strong clinical implications and suggest that much more work is needed moving forward.

### What do we know about immunizing patients with IMIDs for COVID-19?

As of the time of this manuscript preparation professional organizations focused on IMIDs (ie, rheumatologic, inflammatory bowel disease, psoriasis, multiple sclerosis, etc.) are actively deliberating and providing recommendations for providers and patients on vaccine practices, yet they are limited by a lack of evidence in these precise groups. In the clinical trials leading to emergency authorization approval of both mRNA vaccines currently approved in the United States and Europe (Pfizer and Moderna), patients with active IMIDs on immunosuppressive therapies were excluded, and these patients are just now becoming eligible to receive them at the beginning of 2021.

Two areas of consideration for patients with IMIDs are vaccine safety and efficacy. In terms of safety, because these are non-live vaccines, concerns are limited to reactogenicity, allergy, and risk of flaring underlying diseases. The questions of allergic and even anaphylactic reactions to both of the currently available vaccines is based on extremely small numbers of events that have been poorly characterized. Guidance by the Centers for Disease Control and Prevention (updated Dec 17, 2020) (https://www.cdc.gov/coronavirus/2019-ncov/vaccines/safety/allergic-reaction.html) does not specify carrying an IMID diagnosis as a major concern, but focuses on past history of allergic reactions, especially to vaccines, and or components of the vaccines (eg, polyethylene glycol), as well as a history of mast cell disorders. One theoretical area of safety concern among autoimmune diseases are those associated with a Type 1 IFN signature indicating an upregulation of these pathways in immunopathogenesis [84]. This IFN signature has been identified in a variety of autoimmune diseases, but the strongest evidence for its role in immunopathogenesis is in systemic lupus erythematosus (SLE) [70]. In SLE there appears to be an upregulation of IFN signaling that is still incompletely understood, but likely involves both endosomal (ie, Toll-like receptors) and other cytosolic nucleic acid receptors via RNA and DNA triggering [70]. While the currently authorized vaccines have undergone nucleoside modifications, such as pseudo-uridi-nation [85], designed to reduce their capacity to activate nucleic acid sensors in healthy cells, it is unknown whether this will have the same effect in IMIDs such as SLE. Furthermore, in SLE the presence of autoantibodies specific for RNA binding proteins has been demonstrated to amplify IFN production [86], and it is unknown at present whether vaccine mRNA in complex with preformed RNA-binding proteins may ultimately result in immune complexes with resultant pathologic inflammation in such settings. This may be unlikely as autoantibodies are generally not capable of intracellular targeting. A rapid effort to assess these risks is underway, and in the meantime, vigilance for the theoretic potential of these vaccines to induce disease flare in SLE and related disorders must be weighed in deciding whether to vaccinate.

Finally, in terms of vaccine efficacy there are prevalent concerns, modeled on experience with other vaccines, that the diseases themselves and more importantly the therapies for IMIDs may attenuate their capacity to generate protective immunity. Despite this, multiple organizations including the Infectious Disease Society of America [87], and disease-focused organizations all support immunization practices in patients with IMIDs [88]. In patients with IMIDs there is an extensive literature examining the effects of concomitant immune-based therapies, including glucocorticoids, anti-metabolites, targeted therapies including anti-cytokine agents (anti-TNF, anti-IL-6, and others), anti-T cell (abatacept and others) and anti-B cell therapies (rituximab, ocrelizumab, and others) and other novel immune-based therapies on vaccine responses to T-cell dependent, T-cell independent, and neoantigens [88–90]. Not surprisingly, B-cell depleting therapies have been demonstrated to have the most profound effects, which may last for 6 to 12 months or longer or until naive B cells have returned [91]. Anti-cytokine therapies (monoclonal antibodies directed against TNF, IL-6, and others) in the absence of other immunosuppressive therapies have not been demonstrated to significantly inhibit vaccine responses [90, 92]. Among other immunomodulatory agents abatacept (CTLA4-Ig) has been associated with mixed results [90]. Unknown at present is the degree to which these immunomodulatory therapies in the settings of IMIDs will affect both humoral and cell-mediated responses to COVID-19 vaccines. Clinical decisions regarding when to vaccinate in relation to disease activity (ie, quiescent disease versus flare), timing of immunization relative to administration of some therapies, especially B-cell depleting agents, and withholding of certain immunosuppressants such as methotrexate for defined periods around vaccine administration in an effort to augment vaccine response must all be considered [89], but await clinical studies for evidence-based guidance.

### Does autoimmunity contribute to the syndrome of long COVID-19?

The next frontier of COVID-19 is the poorly understood entity of long COVID-19, referring to a heterogeneous group of signs or symptoms experienced in those who have recovered from the acute phase of infection but persist and are not explained by an alternative diagnosis. The clinical manifestations thus far reported are diverse; the most frequently documented include persistent fatigue, dyspnea, brain fog, arthralgia, autonomic symptoms, as well as many others [93]. These patients are often referred to as “long haulers”. While a small fraction may represent the sequelae of delayed recovery from fulminant illness with some shared features of “post-ICU syndrome”, the vast majority appear to occur in patients who had non-severe infection [13]. The clinical pheno-types, risk factors, and pathogenesis remain unclear, although a number of etiopathogenic mechanisms have been proposed, including the unmasking of underlying comorbidities, residual end organ damage from acute/severe infection, persistent or restricted viral replication, and persistent immune activation [15]. As described elsewhere in this manuscript, there have been reports of detecting auto-antibodies post-COVID-19 as well as autoimmune disease, however evidence is lacking regarding causation of the syndrome of long COVID-19. Comparisons can be made between long COVID-19 and myalgia encephalomyelitis/chronic fatigue syndrome (ME/CFS) as many symptoms overlap, and ME/CFS has long been linked to viral infections prior to symptom onset. However, the pathogenesis of both entities remains unknown, as is whether some long COVID-19 patients are suffering from a form of ME/CFS [15, 44]. At present, there is a paucity of data on any role for autoimmunity in long COVID-19 and studies to better understand pathogenesis are urgently needed.

## CONCLUSIONS

The study of COVID-19 in terms of its immunopathogenesis and its relationship to dysregulation diseases of the immune system is providing an unparalleled opportunity for progress in the study of the human immune system. The massive output of this biomedical research effort will likely yield important insights into the immunopathogenesis, treatment, and possibly prevention of diseases of the immune system, including inborn errors of immunity, autoimmune and autoinflammatory, and immunothrombotic diseases among others. The pace of research and output of publications on these topics is unprecedented with nearly 100,000 publications (PUBMED 83,000, MedRxiv 9,300 December 30, 2020) in less than one year. The purpose of this review was not to provide a systematic review of this literature but rather to pose critical questions regarding the intersections of COVID-19 and autoimmunity and attempt to address them with a critical synthesis of select works that may provide insight or direction - at least for the moment.
